# Nature Studies in Macedonia—II

**Published:** 1917-09-22

**Authors:** 


### NATURE STUDIES IN MACEDONIA.—II.

By AN OFFICER IN THE R.A.M.C. AND THE AUTHOR OF "NATURE NOTES."

No one can live in the Struma Valley without becoming interested in the storks. Their fearlessness of man, their conspicuous appearance and their quaint ways have attracted attention to them in many lands, as is shown by the wealth of folklore and fable that has sprung up round them. They are fearless of man no doubt because for generations in all countries they frequent they have been immune from human persecution; but not only are they fearless of man, they seem to prefer to settle near his habitations. It amuses me to wonder what the reflections of these solemn birds are on the change war has brought into their environment. They sit on their old nests and look down on the same villages as of yore, but see a very different race of men surrounding them. Yet still they find themselves regarded friendlily. No man molests them. The only instance of molestation I have heard of was the act of an officer who actually took and ate the eggs from storks' nests. This same officer was also guilty of shooting and eating paired partridges, but he has been taught that neither act is considered conduct becoming a gentleman and a sportsman. It is almost inconceivable that a man of the status of an English officer should have been so lacking in decent feeling as to do such things.

The storks' nests are huge structures, built of sticks, repaired and added to year after year. The foundation, therefore, is often of very old and rotten material firmly pressed down on to the top of some broken tree, the fork of some huge branch, the corner of a roof, or the top of a chimney. Many of them are only a few feet above the ground. The stork lays her eggs on the top of this pile, and on it she and her mate spend much time, sometimes in a leisurely fashion rearranging the sticks, sometimes apparently contemplating their eggs or the scenery, and occasionally indulging in a fantastic sort of game in which they clap their beaks, making a noise very like that made by a boy dragging a stick along a line of iron area railings, and spread and flap their wings. No doubt this is in the nature of song and sexual display. Other birds appear to me to take lodgings in the pile on storeys below the storks. I am nearly certain that sparrows, jackdaws, and starlings all have nests in amongst the lower layers of sticks. I often see an old stork sitting on her nest whilst a starling whistles and works its wings just outside it, sparrows bustle in and out of the sticks below her, and a pair of jackdaws sits in some cranny of the nest pile. One day I will try to get up and examine into this. The male bird, as I have seen, occasionally brings a frog or other dainty and presents it to his sitting mate. I do not know if he takes his turn in incubation.

My duties necessitate long rides daily. I am often out with my horse for eight or nine hours on end. In the course of my journeys I cut across between the beaten tracks over all sorts of marsh and mere and mountain and desolated cultivation. I seldom take my orderly with me because I prefer to be unhampered, able if I wish to stop to watch a bird or a beast without exciting remark. There are few better ways of becoming acquainted with wild creatures than by riding through their haunts. The presence of the horse disarms their suspicions. They do not so much regard the man on its back as they do a man on his own feet. The elevation gives good view, and as the horse is doing all the work of locomotion the rider has nothing to do but to sit quietly as the horse canters along and look about him. In these peregrinations I have endless opportunity to study storks. They are most certainly to be found searching the sides of marshy pools in which are multitudes of frogs, but they are almost equally often on dry grassland and amongst crops. Here they seem chiefly to prey on beetles and, later in the year, grasshoppers of many sorts. A snake, if found, is a welcome prize. I have seen a stork flapping along with a snake nearly a yard long held by the end of the tail; the head dangling down and swaying with the motion of the bird's flight. They strike at beetles very swiftly and unerringly with their great beaks, and swallow them down with ridiculous gusto. I suspect that young birds hatched on the ground would get short shrift. The war has brought some tragedies. Our enemy lately shelled one of our villages, on the houses of which are several storks' nests. On one house a stork was sitting. Two shells went right through the roof, but the bird did not get off her nest; each time she raised her head and gave an indignant "quawk," then settled down again. On another nest there is a stork dead; killed by a piece of shrapnel.

An officer showed me a magpies' nest close to his dug-out in which a pair of kestrels are nesting. The nest is in a young aspen tree and not 20 feet above the ground. This officer had watched the magpies building it till they had got it practically complete. Then two jackdaws took nesting quarters in a pollard willow close by, and proceeded to rifle the magpies' nest for material for their own. Fierce and frequent fights resulted, but after some days the magpies gave up and retired. Almost at once the kestrels took possession, and still fiercer fights took place, in which the jackdaws got worsted and had to resort to more honest ways of obtaining nesting material than using another bird's nest as an R.E. dump.

An officer commanding a field artillery brigade, with whom I used to shoot twenty years ago when we were both subalterns in India, showed me a nest snugly placed in the fork of a poplar tree near his dug-out. I thought it was a hooded crow's nest, and fancied I could make out the beak of a bird projecting over the edge of the nest. We tapped the tree and made noises, but the bird sat fast. There was some chaff, and I said I could still climb up to the nest. Then might have been seen the spectacle of a very senior Staff officer climbing up a tree whilst the officer commanding a field artil-

☛ The first article appeared on August 18.

lery brigade and the bald-headed medical officer of the brigade shoved behind to help him over the first difficulty and roared with laughter. I got to the nest. The "hoodie" slipped off before I reached it. There were four eggs marked like a common crow's, the markings being very much heavier at the large end. The nest was lined with, I think, the hair clipped from some of His Majesty's mules. The O.C. F.A.B. is so fired by example that he has vowed to ascend to a kestrel's nest near by, but he says he can only do it in the morning.

On April 25 I had to go to an advanced position, so I left my horse under cover and walked up with another officer. We made use of a communication trench dug in sandy soil. This trench was full of beetles, chiefly a black sort with short nippers. These beetles are common here, and form a large part of the food of the kestrels. Apparently those in the trench had tumbled in as they wandered on its edge or had emerged from their holes on to its side unexpectedly and so had slipped down. There were many thousands in the trench, some dead, more alive. Some were digging holes for themselves. As we went along many threw themselves into a threatening attitude, raising their bodies so much that some fell over backwards, and distending their nippers widely. The trench was across land that had been cornland last year, but now is uncultivated. It is overgrown with countless millions of wild flowers, a glorious spectacle; the scent of them, sweet as honey, filled our nostrils all the way.

